# The Dying Poor

**Published:** 1871-09

**Authors:** 


### THE DYING POOR.

THERE scarcely beats in any human bosom a heart so hard as to refuse to furnish a dying man, or woman, or child, a little nourishment; that would not cheerfully and promptly give a few spare cents to purchase a cup of soup, or custard, or delicate jelly of some sort. There is not a day in all the year which does not witness the dying of more or less persons in utter destitution in all large cities. There are thousands of tender-hearted women in New York city alone, who, if they knew that any human being within a mile or two of them was within a day or less of death's door, and pining ever so longingly for a spoonful or two of some delicacy, would not ransack their pantries, fill their little baskets with nice things, throw on their bonnets, and without stopping to look in the glass to see if everything was right, would dash out into the street on the half run, not stopping to take breath until the homely dwelling was reached; and others, who had none of these delicacies on hand, would give five times their worth, in the shape of a dollar or two to any one, who, having no money, would be willing to do the personal service himself.

Now, reader, this very moment gather up a liberal sized basket of knick-knacks, or if you have nothing but money, take of either to number 525 West Fifty-first Street, New York city, where resides Miss Sarah Warren, who knows the streets, and houses, and rooms, where the famishing, and sick, and dying are, who need such things, and are needing them this very minute while you are reading this sentence, and who, if they have not the help this day, will not need it to-morrow, because to-morrow they will be dead. Miss Warren has young ladies, and widows, and married ladies, and men, who take these delicacies to the sick every day, and have been doing it for a year, and expect to do it for a year to come, for the very love of it, without money, and without price. The names of some of the persons engaged in this humane work are, Frederick Wright, 10 West 36th St.; D. I. Reynolds, 57 West 28th St.; W. Curtis, 39 West 50th St.; Mrs. T. Carmichael, Mrs. Hunting, Mrs. C. A. Taylor, Mrs. A. M. Hays, Mrs. J. B. Kissam, Miss Turnbull, Miss J. E. Turrill, Miss Sarah Start, Mrs. Stewart, Mrs. Fitzsimmons, Mrs. Adams.

This Society helps all persons, without regard to race, color, or creed. All the persons named perform their work without any compensation whatever; the only expense incurred is the rent of the house from which things are kept to be prepared and sent out; thus every dollar contributed goes directly to the relief of the suffering.

When poor women are found who cannot earn enough to feed themselves, such persons are furnished for one or two days in the week with a bowl of good soup at the house. During the last year two thousand persons were assisted in this way at an expense of less than six hundred dollars.

Who of our readers will give money, who provisions, who themselves? If money, send it to the Treasurer, H. W. Curtis, 39 West 50th St. If provisions, to Miss Warren, 525 West 51st St. If yourself, report to the Lord; "Here am I, send me."

The Editor does not know whose head, and heart, and hand put such a charity in operation, but it is just like "Young Tyng," son of "the noblest Roman of them all."

